# Personal and organisational health literacy in the non‐specific symptom pathway for cancer: An ethnographic study

**DOI:** 10.1111/hex.14062

**Published:** 2024-05-05

**Authors:** Georgia B. Black, Julie‐Ann Moreland, Naomi J. Fulop, Georgios Lyratzopoulos, Brian D. Nicholson, Katriina L. Whitaker

**Affiliations:** ^1^ Centre for Cancer Screening, Prevention and Early Diagnosis, Wolfson Institute of Population Health Queen Mary University of London London UK; ^2^ Department of Applied Health Research University College London London UK; ^3^ Department of Radiology Oxford University Hospitals NHS Foundation Trust Oxford UK; ^4^ Department of Behavioural Science and Health University College London London UK; ^5^ Nuffield Department of Primary Care Health Sciences University of Oxford Oxford UK; ^6^ Department of Cancer Care, School of Health Sciences University of Surrey Guildford UK

**Keywords:** cancer, early diagnosis, health literacy, patient communication, qualitative

## Abstract

**Introduction:**

People being investigated for cancer face a wealth of complex information. Non‐specific symptom pathways (NSS) were implemented in the United Kingdom in 2017 to address the needs of patients experiencing symptoms such as weight loss, fatigue or general practitioner ‘gut feeling’, who did not have streamlined pathways for cancer investigation. This study aimed to explore the health literacy skills needed by patients being investigated for cancer in NSS pathways.

**Methods:**

This study employed ethnographic methods across four hospitals in England, including interviews, patient shadowing and clinical care observations, to examine NSS pathways for cancer diagnosis. We recruited 27 patients who were shadowed and interviewed during their care. We also interviewed 27 professionals. The analysis focused on patient communication and understanding, drawing on the concepts of personal and organisational health literacy.

**Results:**

Our analysis derived six themes highlighting the considerable informational demands of the NSS pathway. Patients were required to understand complex blood tests and investigations in primary care and often did not understand why they were referred. The NSS pathway itself was difficult to understand with only a minority of patients appreciating that multiple organs were being investigated for cancer. The process of progressing through the pathway was also difficult to understand, particularly around who was making decisions and what would happen next. The results of investigations were complex, often including incidental findings. Patients whose persistent symptoms were not explained were often unsure of what to do following discharge.

**Conclusion:**

We have identified several potential missed opportunities for organisations to support patient understanding of NSS pathways which could lead to inappropriate help‐seeking post‐discharge. Patients' difficulties in comprehending previous investigations and findings could result in delays, overtesting or inadequately targeted investigations, hindering the effective use of their medical history. Third, patients' limited understanding of their investigations and results may impede their ability to engage in patient safety by reporting potential care errors.

**Patient or Public Contribution:**

Patient, public, clinical and policy representatives contributed to developing the research objectives through a series of meetings and individual conversations in preparation for the study. We have held several events in which patients and the public have had an opportunity to give feedback about our results, such as local interest groups in North London and academic conferences. A clinical contributor (J.‐A. M.) was involved in data analysis and writing the manuscript.

## 1 INTRODUCTION

It is now recognised that patients play a crucial role in their own healthcare journey. The paradigm of patient‐centred care emphasises the importance of involving patients as active partners in their treatment decisions and safety.^1^ As part of this concept, the National Health Service (NHS) Patient Safety Strategy has highlighted patient understanding as a key element in enhancing patient engagement and safety,^2^ particularly as a way of preventing errors in continuity of care.^3^ The strategy aims to empower patients and their families to become vigilant stakeholders in safety, transitioning from passive recipients of care to active participants. Central to this transformation is the understanding that patients' comprehension of their care profoundly impacts the quality of care received and the overall clinical outcomes.

The ability to understand and use health information to make informed decisions is a critical factor in patient engagement and safety.^4^ Research has indicated that patients who understand their care tend to have better clinical outcomes and improved quality of care.^5^ Conversely, poor understanding has been associated with specific adverse clinical outcomes across various medical conditions.^6^, ^7^, ^8^ The term ‘health literacy’ incorporates both *personal* skills needed by individuals to navigate the health system and understand health information, as well as *organisational* efforts to communicate effectively and provide information in a way that supports patient understanding and engagement.^9^ Personal health literacy skills include knowing when and where to seek health information, retaining and processing information and being assertive.^10^ Health literacy has been found to have a more substantial impact on outcomes than socioeconomic factors such as race, income and education,^5^ although these vulnerabilities co‐exist and interact.^11^ Recognising the critical role of patient understanding of their care, global health organisations such as the World Health Organization have highlighted health literacy as a vital quality and safety concern.^12^

Relatively little information exists about how patient understanding and health literacy affect the timely diagnosis of symptomatic cancer, with some indication that processes of monitoring and evaluating symptoms may affect the time to diagnosis.^13^, ^14^ Cancer, being one of the most complex and challenging diseases, presents unique difficulties for patients in comprehending diagnostic pathways and treatment options. Studies have shown that patients often face significant challenges in understanding cancer‐related information, associated with suboptimal care.^15^, ^16^, ^17^

Enhancing organisational health literacy is an essential component of clinical practice in cancer diagnosis, as even highly health‐literate individuals can struggle to understand and act upon cancer‐related information.^16^, ^18^, ^19^ Poor organisational health literacy can result in lower standards of care and diagnostic errors in communication, care coordination, care access and record accuracy.^20^ Multiple studies have demonstrated that contextual factors such as the service environment and structural barriers are critical to the quality of clinician–patient communication.^21^, ^22^

**Figure 1:**
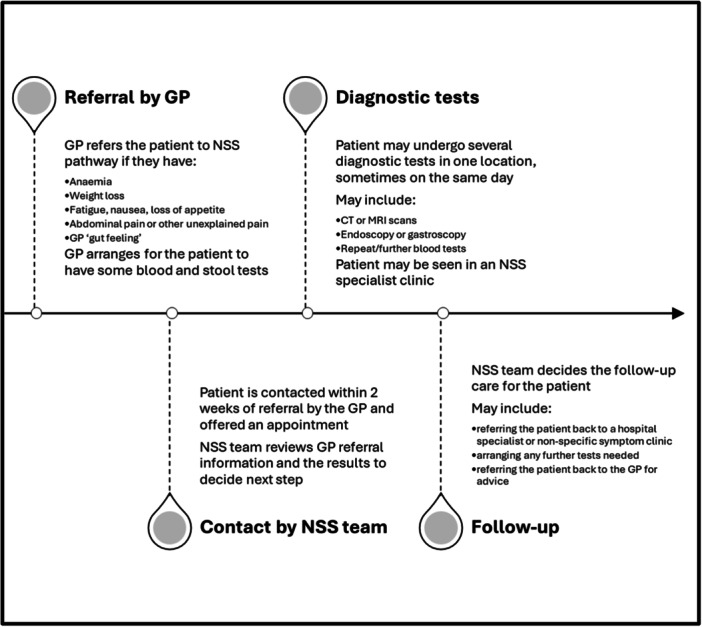
The NSS pathway model. GP, general practitioner; NSS, non‐specific symptom.

Non‐specific symptom (NSS) pathways were implemented in the United Kingdom in 2017 to address the needs of patients experiencing symptoms such as weight loss, fatigue, anaemia, abdominal pain or general practitioner (GP) ‘gut feeling’, who did not have streamlined pathways for cancer investigation.^23^ The pathway is shown in Figure 1.

Patients are holistically assessed and investigated, rather than seeking to exclude cancer from one particular organ. As part of their design, NSS pathways adopt a patient‐centred approach which prioritises patient communication, with dedicated patient ‘navigators’ to facilitate timely communication and access.^23^, ^24^ However, evaluations to date have not focussed on patient understanding of these pathways and the impact on the quality and safety of patient care. This study aimed to explore the health literacy skills needed by patients being investigated for cancer in NSS pathways.

## 2 METHODS

This paper draws on a variety of data sources collected as part of an ethnographic study^25^ in NSS pathways, including interviews, patient shadowing and observations of clinical care (see Table 1). The aim of the full ethnographic study was to explore diagnostic safety in NSS pathways to inform healthcare improvement. This paper presents part of the findings. Initial ideas relating to the development of this study were discussed with a focus group of patient representatives, who highlighted the difficulty of managing and navigating healthcare in the context of NSS. The study received ethical review and approval from the East of Scotland Research Ethics Committee (21/ES/0076).

**Table 1:** Patient participant demographics.

Sex	
Female	13
Male	14
Age	
Mean	67.6
Range	35–94
Highest level of educational attainment	
None	3
Ordinary level/General Certificate of Secondary Education or equivalent	3
Advanced level or higher	5
Higher education qualification below degree level	9
Degree	6
PhD	1
Employment status	
Working full time	8
Working part time	5
Retired	14
Self‐reported ethnicity	
White British	17
White (other)	2
Black African	1
Asian	3
Asian British	1
Black Caribbean	1
Marital status	
Single	5
Married	14
Divorced or separated	3
Widowed	5
Index of Multiple Deprivation quintile by patient postcode^26^	
1 (most deprived)	1
2	6
3	6
4	8
5 (least deprived)	6

### 2.1 Participants

#### 2.1.1 Patient shadowing

Twenty‐seven patients undergoing NSS pathway investigations participated in the study through either shadowing or formal interviews (Table 1). All patients who had been referred to the NSS team were eligible to be approached by an NSS staff member and taken through formal informed consent processes. Patients with all types of NSS were eligible to participate. Patients were recruited to obtain a diverse sample: we sampled patients from a wide range of health experiences and demographic characteristics such as age, gender, education, socioeconomic status and geographic location. Due to the broader focus of the full ethnographic study, we did not assess patients' health literacy or sample explicitly for this. Other studies have shown that predictors of health literacy include age, income, race, education, employment and physical and mental health status.^27^, ^28^, ^29^

#### 2.1.2 Interviews

A total of 27 professionals were interviewed to gain insights into their perspectives and experiences of NSS pathways (Table 2). All staff working in NSS pathways were invited to participate in the interviews. One declined, and two were unable to participate due to competing pressures. Staff working in Cancer Alliances or commissioning roles were identified through NSS networks and were invited to participate via email (*N* = 7). Additionally, policymakers in the field of early cancer diagnosis were interviewed (*N* = 3) to gather insights into relevant policy directions in new diagnostic pathways, the impact on practice and the significance of patient safety within cancer referral pathways. Recruitment of policymakers was facilitated through contacts in the NHS Cancer Programme (NHS England).

**Table 2:** Professional participant employment information.

Professional role	
Healthcare providers	
Consultant	*9*
Nurse	*4*
Navigator	*3*
Cancer alliance/commissioners	9
Policy maker	3
Context	
Site 1	3
Site 2	2
Site 3	4
Site 4	8
National	2
Cancer alliance 1	4
Cancer alliance 2	3

The recruitment process also followed the principle of purposivity, which involves selecting participants who could provide valuable insights and perspectives about their involvement in NSS pathway investigations. This approach allowed for the inclusion of individuals who had firsthand experiences with the healthcare system, referrals and various aspects of patient care within the context of NSS.

### 2.2 Data collection

#### 2.2.1 Observations

The study involved data collection from four NHS trusts between March 2022 and January 2023. The sites were selected through local healthcare networks, mindful of choosing sites with contrasting service design, clinical leadership and population characteristics. A multisite design was employed, incorporating both in‐person observations in the hospital and online staff meetings through Microsoft Teams, spanning a total of 43 h. The observations aimed to capture various aspects of everyday practice, including patient‐facing clinics, staff meetings and desk work. We took an exploratory approach with the researcher (GBB) engaging NHS Trust staff to understand the relevant environments for the study. The researcher was experienced in qualitative interviewing and observational data collection. Throughout the observation phase, the researcher took unstructured notes on‐site, which were transcribed and expanded in digital form. This typically included a chronological account of the observation, including relevant people who were present, short extracts of conversation and procedures and equipment that were used. It also included questions or points for future clarification, and reflections about the impact of the researcher being in the environment. We also collected pertinent documents such as policy documents, referral forms, business cases and meeting minutes that were specifically related to NSS pathways. This document collection was a combination of responsive actions, where local staff members provided the documents, and proactive measures, such as targeted internet searches for relevant guidelines or reports, and requests for confidential document sharing. Relevant documents to this paper include:

1. 2019 Rapid Diagnostic Centres Vision and 2019/20 Implementation Specification.^30^
2. 2022 NHS Faster Diagnosis Framework.^23^

#### 2.2.2 Interviews and patient shadowing

Shadowing and interviewing were conducted by G. B. B., a senior qualitative researcher with extensive experience in cancer diagnosis. All patients were invited to participate in at least one in‐depth formal interview, during which open‐ended biographical questions were asked regarding their experiences in primary care, referrals, clinical contacts and any other relevant perspectives or experiences (see supplementary material). Fifteen out of the 27 patients were also shadowed over time within a network of healthcare settings, including multiple hospitals and primary care. Shadowing typically started after the initial interview and ended when the patient was discharged from the pathway. The reason some patients were not shadowed was because we were not able to schedule the first interview until they had already received their test results and been discharged from the pathway.

Shadowing activities were conducted in person, such as waiting for clinic appointments or tests, as well as through brief telephone interviews or email contact to gather recent event information. This typically included details about recent procedures and patients’ expectations about what would happen next. This approach aimed to encompass not only the doctor–patient relationship but also the structural and cultural factors influencing patient care experiences such as waiting for tests or results, access to and comprehension of information and breakdowns in communication between hospitals and primary care. The longest period of shadowing was 1 month. None of the participants interviewed were diagnosed with cancer during the study.

Professional interviews were conducted by G. B. B. and one other experienced qualitative researcher. The staff, cancer alliance/commissioner and policy maker interviews followed a semistructured format, focusing on issues related to their perspectives on NSS pathways, their understanding of particular concerns and safety issues for patients and areas they identified for improvement (see supplementary material). This included questions about NSS pathway design, what would indicate that the pathway was working well and any particular concerns or issues with patient safety. These interviews specifically addressed diagnostic safety issues in cancer referral pathways, including the integration of the NSS pathway into local cancer services.

### 2.3 Data analysis

The analysis followed an iterative process of inductive coding and exploration of the complete data set, informed by a patient safety framework.^31^ The framework encouraged attention to a wide range of influences on clinical care including institutional, organisational, financial, cultural, staffing, team and patient factors. The research team continuously met during the fieldwork, allowing for the development of ideas and explanations as the study progressed. Having completed the inductive analysis, we focussed our attention on codes relating to patient experience, communication and understanding of the service. We refined the codes by re‐examining both extracted quotations and whole transcripts and revisiting observation notes.

Following the completion of coding, we noted that several elements relating to patient understanding and communication had been generated. We considered how health literacy concepts (such as knowledge, interactive and judgement skills)^32^, ^33^ might deepen our understanding of the data, although the aim of our ethnographic study had been to understand the patient safety aspects of the NSS pathway. Other aspects arising from our analysis will be published in further papers. Therefore, our focus in this analysis was to elucidate the particular health literacy demands of the NSS pathway, rather than cancer investigation or healthcare more generally. We also do not report here on information‐seeking or interpersonal communication outside the health system which would be relevant to health literacy more widely.

These findings were critically evaluated by patient, public, clinical and policy representatives at several stakeholder events.

### 2.4 Findings

Our findings revealed considerable difficulties faced by patients in understanding the NSS pathway, along six thematic stages:

1. The work up in primary care.
2. The reason for referral.
3. The nature of the NSS pathway.
4. The process of investigation.
5. The findings of tests and investigations.
6. The meaning of unresolved or unexplained symptoms.

We found personal health literacy skills to be important in understanding information, interacting with staff and making complex judgements about ongoing care. Organisational health literacy was mixed, with variable communication and support in relation to complex medical concepts and decisions. However, the importance of patient understanding was included in the 2022 NHS Faster Diagnosis Framework, which outlined the specification for NSS pathways: ‘All communication with patients and carers is presented in a way that they will understand, taking account of language, cultural, sensory, learning or other needs’.

### 2.5 Primary care work up and investigations

Most patients (*n* = 19) described a thorough work‐up process by their GP that led to an NSS pathway referral, requiring personal health literacy to understand a wide range of tests and interventions. Depending on the patient's symptoms, this included blood tests (which were then discussed in primary care), direct chest X‐rays, prescription medications (e.g., pain relief, iron), urine tests, stool tests and so on. Some patients had been referred to local investigations units, for example, haemophilia unit, ultrasound clinic. Sometimes the multiple investigations were hard for the patient to keep track of and make sense of:

I've had stool tests and urine tests, an X‐Ray was done, and it all came back negative, they said there was nothing wrong with me. And I had ages ago, there was a low level anaemia, but that was never addressed with the doctor, just seemed like it didn't matter.[…] I went to a chest X‐Ray. Each time they've said, ‘Well, there's literally nothing wrong with you’. Low level anaemia was something that was mentioned but I don't even know what that is. (05H1, Patient)

Most patients had been seen in person, and many had been physically examined. This was frequently cited as the reason for referral, where physical findings such as tenderness provoked the motivation for referral. A significant minority (*N* = 8) of patients were referred at the initial consultation, without any previous investigations by the GP. These patients tended to be characterised by presenting with weight loss. One nurse suggested that this may be because weight measurement was done ad hoc in primary care, often without further investigations or discussions about the cause of weight loss:

Some patients would go into their GP for something totally different and the GP will weigh them and say okay you have lost weight without asking the patient, going through because they have limited time. Without going to more in depth with the patient because those patients told me a lot of things that is happening that is going on with them, sometimes they have lost their mother, their father, their brother, their sister. But the GP has referred them for weight loss because they haven't been through with the patient or whatever, they don't have time to go through. (RDC14, Nurse)

A small minority of patients were referred without seeing the GP in person (telephone consult). This was sometimes due to unusual blood test results or findings from another investigation, which created a need for interactive health literacy, assimilating information from multiple sources:

I didn't see a doctor, but I had a conversation, and I can't remember her mentioning that there'd been a report. I had been examined, but there'd been no report about me being anaemic, and I think this might be, might be the fact that I perhaps, I should have contacted somebody, and I didn't. I wasn't aware that I was supposed to contact someone. (20H4, Patient)

### 2.6 The reason for referral

Just over half of patients (*n* = 16) were investigated by other specialist services before being referred to the NSS pathway. This included lung, colorectal, hepatology, upper gastroenterology, gynaecology and cardiovascular pathways. In some cases, this was a targeted approach due to specific symptoms such as difficulty swallowing or swollen legs; in other cases, patients reported a broader approach by GPs to investigate multiple organs as the patient's symptoms persisted or worsened. For patients who had had multiple previous investigations, it was often interpreted as a ‘last resort’ where other services had failed to find the cause of the problem. For some patients, this was a relief, feeling that things had ‘gone on too long’ and that this would be a positive step to try and understand the cause of their symptoms. For other patients, the number of tests and investigations were burdensome, and some patients felt that the referral was made as a ‘last resort’ rather than a logical step.

Well I think it's—it seemed to be because they'd exhausted everything else. And it was another tick to put on the box you know, rule it out, and I figured that's why it happened. Because I wasn't expecting that so when I got the letter from the hospital saying that I'd been called in for this, and I saw the word cancer on it, it kind of alarmed me, because I didn't think it was that, you know among the various things I was wondering what it might be, so yeah. (15H4, Patient)

Patients were frequently aware that a blood test result had provoked the referral without necessarily knowing that this could indicate a potential cancer. For example, one patient mentioned having an unusual potassium level which triggered the referral.

She seemed to be worried about the anaemia, because I was so anaemic. […] And my potassium level, because‐ I mean, she's being good. I've seen her again. She said my potassium level was okay now. (20H4, Patient)

### 2.7 The nature of the NSS pathway

Patient understanding of the NSS pathway was highly varied, suggesting a lack of organisational health literacy in primary care, with some GPs unable to accurately describe the nature of the service to the patient, or that patients lacked the personal health literacy skills to ask about it. Not all patients were aware initially that they were being referred to a cancer service, and very few knew that it was for NSS. These patients' understanding was mainly that they would have ‘further testing’ or that the aim was to get to the bottom of particular symptoms.

They just said that they were going to refer me to further testing. And, yes, once again I did not pick up from the beginning that it was going to be so focused on the big C word, and I actually do appreciate that because they don't want to freak me out more so than I already am. So that's kind, but they had already mentioned that they're quite concerned, that they're 100% persuaded that there's an issue going on here because they have seen my shaking. They have seen my sweating, and, yes, they also are scratching their heads because it's not just one. It's two. They're also scratching their heads thinking, We have got to, you know, somehow identify what is causing this because this guy is suffering. So any kind of possibility I think they're pursuing. (06H2, Patient)

Some patients reported that they did not know what type of service they were being referred to, with a more generic understanding that it was just the next step. Several patients reported being told that they were being referred specifically for tests (and particularly a ‘scan’) without reference to what was being investigated or by what type of specialist team.

We were having a conversation. I mentioned I've been trying to eat more. So, she said, ‘Okay, well jump back on the scales’. And that's when she realised my weight had gone down from the first‐ Two months ago, when I'd gone. And an alarm bell triggered, I presume, and she said, ‘Right, I'm going to send you for this scan’. (05H1, Patient)

Patients who *were* aware of being referred for a suspected cancer did not seem unduly concerned, but rather reassured that their symptoms were being thoroughly investigated. However, these patients did not seem to be aware that they were being referred to a team which could investigate multiple organ systems.

I think from what she was saying initially, it was just because I had said the chronic fatigue had been bad the whole of this year, well, I think we'll just have a look, and then when she weighed me, she did all sorts of other tests; she did a breast examination an internal examination to check, but she couldn't see anything there. She said we'll do it just to rule out that there isn't cancer or anything, it's the weight loss that was a red flag for her. [INT: Did she say what kind of service it was or what kind of doctor you might see?] No, she just said it was an urgent referral and they would probably want to do a scan or something like that just to check out that there weren't any nasties. (26H3, Patient)

A small minority of patients (*n* = 6) were aware that it was a cancer service that would give rapid diagnostic advice and/or investigate multiple organs, and one patient was aware that the NSS service could refer them to another specialist:

I've got a feeling that it was trying to bring everything under the same roof so that if the scan finds something there, then instead of me having to lump it all over the place looking for the right person to deal with it, the pathway would quickly point me, or point the various experts to me. It will then seem like seamless sort of care, because at the moment it's so disjointed. I get one thing checked and go somewhere else and get something else checked. And I'm thinking—but I'm dealing with the one NHS service [laughs], it's all so disjointed. And it would be nice if someone said, ‘Right, let's check this out, and if there's something there we'll refer you there and get that checked out’. And then you know that you don't have to sort of fight your way though each step of the process. (15H4, Patient)

One navigator reported that they had trained the administrator to give patients more information at the point of booking their first appointment, which had reduced patients’ confusion about the service:

I think the fact is that [administrator] will give the patients a bit of a heads up when she calls them. She is kind of their first point of contact, so they have already had that conversation with her. And I have definitely noticed since she started the conversations that we have are easier because they will say oh I spoke to the lovely lady before and she has told me that kind of thing. […] ‘oh no Doctor Bloggs didn't tell me I was having a scan, they didn't tell me why I am having it’, I very rarely have those conversations now. (RDC17, Navigator)

Generally, patients who were aware of the nature of the NSS pathway were appreciative, recognising that this was a faster way to eliminate multiple potential underlying causes for their symptoms.

### 2.8 The process of investigation

Once patients had started being investigated by the NSS pathway, there was often a lack of clarity about who was making decisions about care, and what would happen next. We observed personal and organisational health literacy skill deficits in that staff did not often communicate this explicitly to patients, and patients seemed unaware that this was something they could ask about. For example, several patients did not know that a consultant had reviewed their referral and was contributing to decisions about their care. One patient thought that it was the GP who had ordered the scan and was not aware that a referral had taken place (‘Nothing's been done through the hospital; it's all been arranged through the GP’. 05H1)

In one clinic observation (H3), we witnessed an encounter that exemplified patients' difficulties with understanding the process of referral. The patient came in with his daughter and the clinic doctor asked the patient ‘What tests have you done?’ which caused some confusion. She tried a different tactic: ‘What did you say that worried the GP?’—again, some confusion. The daughter claims that her father went to a diabetes clinic and the nurse wrote his weight down wrong, according to the daughter. ‘Better to be safe than sorry’ says the doctor. However, the referral form gave a different reason: the patient has raised calcium in his blood tests. The daughter mentions that they were also worried about his blood pressure results in the waiting room as her mum/the patient's wife came into the hospital last year with high blood pressure was discharged home, and had a heart attack soon after.

After getting dressed, the patient reported having had a computed tomography (CT) scan at the Whittington last month but it was not clear why. The daughter explains that her father has routine checks annually because of his job. There is also some discussion about a throat problem, and also a chest X‐ray. The doctor suggests that they will not repeat the CT scan if he has had one recently. The daughter seems confused about what a CT scan is and asks ‘is it the one where you stand up?’—the doctor explains that no, it is the one where you lie down. So the patient has only had an X‐ray after all, and there is still a need for a CT.

Our data collection procedures were designed to capture different contact points in the patient pathway; patients sometimes had to wait for several days and in a minority of cases, over 2 weeks to find out the test results. Patients were often unsure about what would happen next in the pathway; for example, whether they would need further tests or how they would be contacted.

To be honest, I didn't expect, if there's nothing wrong, I don't expect to hear anything. I think I'd only hear something if something's wrong, so‐ I mean it would be nice to hear from somebody, just to sort of say, ‘Oh, everything was fine. There was nothing’, you know, whatever. But I think everybody is so busy now that you probably won't hear unless there is something that shows up, so I'll just sit tight and wait and see. [INT: Yes, yes. So, do you have any idea who's going to be thinking about that for you?] Well, I presume the people that sort of started it all off. The Rapid Response people will have been sent a copy of the CT scan, and I imagine that those people at the [hospital 1] will be looking into it, but you know, I'm hoping they will be looking at it anyway. (12H1, Patient)

Patient navigators were particularly important in terms of managing patients' information needs in the pathway. Many patients particularly appreciated that navigators would provide information about how results would be communicated:

She said she would ring me and say what the results of this scan are, then she will decide what next to be done next if anything and I'm sure she will send a copy of whatever she is going to do to the doctor I guess. I haven't been back in touch with the surgery because I'm patiently waiting for this scan to see the way forward. (23H4, Patient)

Patient navigators recognised that waiting for results was a difficult part of the pathway for patients, and tried to help them to anticipate what was going to come next:

You can also anticipate the level of detail in terms of the results that you will get and how helpful that will be. And then you can also anticipate like the next test and being able to explain to patients why they have to have another test in a way that's not sort of fluffy and be like oh well we weren't quite sure on the CT so we are going to look at MRI. For some patients that's enough but for actually quite a lot of them that's not quite the message that they need. (RDC16, Navigator)

### 2.9 NSS pathway investigation findings

The 2022 NHS Faster Diagnosis Framework highlighted that patient understanding of diagnosis was particularly important (p. 35). While patient navigators in the NSS pathway worked hard to communicate findings clearly to patients, it was not always effectively received. There were some specific difficulties associated with the NSS pathway that hindered patient understanding. First, the predominance of chest‐abdomen‐pelvis CT scanning resulted in a large number of incidental findings or diagnoses. Mostly, these were reassuring and required no further treatment. However, some patients lacked personal health literacy skills to understand new medical terminology as well as the consequences of ongoing investigation or treatment. This could be distracting especially when there was a lack of immediate relevance to their care:

What I remember, right, I've got a bit of trouble with the spleen, but I knew that before. I think it's called venous malfunctions or something which doesn't really mean much to me but they all seem to be benign which I suppose is the main thing. What else was there? I've got a fatty lump thing on my shoulder but I've had that for years and it hasn't changed at all so that was okay. I've got bronchiectasis or something, I can never pronounce it at the bottom of both lungs. (23H4, Patient)

Some patients found the incidental findings quite anxiety‐provoking:

They just said you have got some, what do they call it, inflammation of the stomach. Yes, yes. Some inflammation, yes.[…] Yes, yes, it put a bit of worry in my head, yes. But after I had, you know the scan, I think it cleared a bit of my head, yes. They said, ‘Everything is okay’, yes. But just this 10% only now that's still there, yes. (19H2, Patient)

In observations, we witnessed the complexity of the information being transferred. For example, we observed a clinic (site H4) where a consultant was conveying the results of a patient's CT scan. The patient came in and they started to discuss the results. The doctor asked, ‘Did you understand the scan?’ The patient said that she did, but that it was complicated. There was a swelling in the small bowel and the tubes to the kidney were thickened/narrow. The doctor explained that she would need a different scan to look at the ureters (tubes) using contrast to ‘create better pictures’. The doctor and patient then discuss her symptoms and go through a physical examination. The doctor suggests a further kidney scan and an endoscopy. The patient is reluctant to do the endoscopy and said that she had had one before and had to have sedation. She could not remember why she had the endoscopy. They discussed some alternatives and the various advantages and disadvantages of the options for further investigation. Finally, the patient asks ‘Could I still have cancer?’. The doctor says that it is still possible, as some areas of the body, particularly those like a tube (e.g., oesophagus) are not visible on a scan. The patient is a bit upset, as she had been reassured by the initial clear scan, and thought cancer had been ruled out. The doctor comforts her with the knowledge that cancer is less likely given her test results. He concludes, ‘there are many different explanations for what you're experiencing’.

### 2.10 Unresolved or unexplained symptoms

Most patients were reassured by NSS pathway findings and felt that they had an explanation for their symptoms. However, a significant minority of patients (*n* = 10) required additional judgement skills to assimilate the meaning of their persistent, distressing symptoms with the advice and results from the NSS team. Most of these patients returned to their GP shortly after being discharged from the pathway and were concerned that the NSS pathway might have exhausted other avenues of investigation:

I really don't understand it because if the hospital can't find anything, I don't know what my GP will say, I don't know how that's going to work out. So, I mean there's nothing much anyone can do, you just do what you're told, isn't it? [INT: No, okay. You sound a bit concerned still.] Yes, [I am still] in pain. (04H1, Patient)

One concern voiced was that having been discharged from a cancer pathway, their case would be deprioritised by primary care. Patients considered that they needed to assimilate the NSS pathway discharge notes and reform a new case for prioritisation in primary care:

But now I'm back to‐ not being rude, back in the system that nobody cares. So I'm just back on a pile where nobody's doing anything again. So, as fantastic as the cancer team were, I'm now just in a waiting system that is completely overloaded because the NHS is, and I'm sitting here suffering for God knows how long again. So, I'm just back to where I started. (17H4, Patient)

The presence of persistent symptoms was a motivating factor for patients in urging their GP to make further referrals and causing worry. Some patients displayed judgement skills, for example, in worrying that something had been missed on a scan due to a lack of contrast agent (a compound used to enhance the visibility of internal structures):

I mean they have eliminated I hope, lung cancer… my GP was frank and said we want to make sure it's not cancer, you know I do worry a bit that the… I didn't have a contrast agent you know they might have missed something, the CT scan might have missed something. (03H2, Patient)

NSS pathway staff were also disconcerted by discharging patients whose symptoms could not be explained. Some staff tried to support patients by creating an opportunity to be rereferred if the patient's symptoms persisted over a long time:

We tried to, then that's where we kind of appeal for patients reactiveness you know and look after themselves and their own health. We always say like, you know if, continue if it's a weight we say please continue your dirty, once a week weight monitoring and if you continue to lose weight you know in the next three months you know go back to your GP and ask them to be referred back to us and we are happy to do more investigations and try to figure out what is happening. It's hard when we can't really find a reason for it. (RDC03, Nurse)

Some NSS pathway staff reported that they would not discharge a patient if they had any concerns that something had been missed or had not been satisfactorily investigated, reducing the burden on patient health literacy. This could involve a period of waiting while the patient was retained by the service:

When my spidey senses are up and I really think there is something there, I might delay them for three months and go, well there's nothing there I know that you're obviously still losing weight, and there's other things going on, but at this moment we have not got any other tests to do. You know we've tested your pancreas, we've tested your upper GI, we've scanned everything you know a PET scan, but I hear things are not right but I don't know where else we can go looking. (RDC20, Consultant)

Others made suggestions to the patient about alternative avenues for support, such as self‐referral to counselling.

## 3 DISCUSSION

This study has highlighted the considerable difficulties faced by patients in understanding the organisation, clinical roles, medical tests and investigations and outcomes associated with the NSS pathway. Despite this, many patients were satisfied with their care and relieved to have cancer ruled out. In particular, it highlights the complexity of the information being conveyed by multiple clinicians at every stage of the NSS pathway: patients struggle to understand why they have been referred since their NSS are often experienced in constellation with other more prominent symptom experiences. We cannot know whether GPs mentioned that they were referring patients to a cancer pathway—our findings show that patients did not always understand this. Both the complexity of the investigations and their results were hard for patients to comprehend, with the additional burden of incidental findings. Patients who were discharged from the pathway without an explanation for their (often long‐suffered) symptoms reported feeling particularly disheartened.

Poor organisational and patient health literacy associated with the NSS pathway could result in a number of adverse outcomes for patients. First, most patients were unaware of the comprehensive and specialist nature of the team they had been referred to (and why they had been referred to them), which is a significant missed opportunity for reassurance and informed decision‐making, and may result in inappropriate help‐seeking following discharge. Second, patients' difficulties in understanding previous investigations and findings could result in delays, overtesting or inadequate targeting of investigations as patients struggle to be bearers of their own relevant medical history. Third, patients' lack of understanding of their investigations and results may hinder their ability to participate in patient safety by notifying staff of any errors in care.

### 3.1 Relevance to existing literature

It is common for patients to find medical information difficult to understand and hard to remember.^34^ Our study suggests that NSS pathways require understanding and remembering of perhaps a greater amount of information than other cancer pathways, due to the complexity of patients' presenting problems and the strategies for investigation; this is likely to challenge correct recall of relevant information.^35^ This is also likely to be socially patterned, as previous studies have demonstrated that patients with lower health literacy feel less informed about cancer testing.^36^ Additionally, NSS pathways may introduce higher patient anxiety through the increased uncertainty inherent in non‐specific symptoms.^37^, ^38^, ^39^

Our results showed that the NSS pathway made significant health literacy demands. For example, some patients did not know that they were on a cancer pathway; experimental studies have shown that patients are more likely to be active participants in patient safety if they perceive their health to be threatened.^40^ The inclusion of ‘gut feeling’ as a criterion for NSS referral may mean that GPs see it as a general means of further investigation rather than cancer‐related per se.^41^, ^42^ We found that patients required skills to independently track and interpret test results to make informed decisions about further help‐seeking, particularly where unresolved symptoms persisted. This is a novel contribution to the area of health literacy during diagnostic work up, where a systematic review of the influence of health literacy on the timely diagnosis of symptomatic cancer found only three papers.^13^ The authors noted that patient engagement with information on cancer symptoms, risk factors and diagnostic tests impacts their awareness and willingness for investigations. However, the review found no evidence relating to health literacy skills required during diagnostic testing for cancer.

Many patients in our study felt well informed by navigators, and the navigators were, by turns, aware of patients’ issues with understanding the pathway. Other studies suggest that patient navigators particularly improve care for vulnerable patient groups who tend to underuse health services, with noted improvement in process outcomes.^43^, ^44^ In cancer care, patient navigators have also been shown to improve the quality of care for more deprived patients and to increase adherence to diagnostic follow‐up care.^45^, ^46^ This adds to the body of evidence suggesting that multilevel interventions will have the greatest impact on cancer diagnostic outcomes, including components such as clinical communication training, navigation, patient empowerment and reducing barriers to access.^47^, ^48^ A systematic review of health literacy interventions in cancer found 36 unique interventions, but none of them were designed for the diagnostic interval.^49^

### 3.2 Strengths and limitations of the study

This ethnographic study drew on multiple sources of data including observations and interviews with a wide range of participants. Drawing on these rich data courses enabled a greater contextual understanding of both patients’ informational needs, the context in which it was being experienced, and the repercussions on patient care. Relatively few staff were recruited at one site; this is due to the size of the team. However, this may have given greater weight to the experiences of staff working on the sites with bigger clinical teams.

We have drawn inferences about patients' understanding and the impact on their quality of care; however, we did not seek to investigate health literacy or sample our participants with health literacy in mind. This is a limitation of this paper. Our sample was relatively well educated with 79% completing secondary school education, which may have limited our interpretation of patients' health literacy skill use and understanding. However, our findings incorporated a wide range of views and observations that increase the rigour of our interpretations.

### 3.3 Implications for practice and policy

The patient‐facing roles in NSS pathways are particularly important to addressing patient understanding, and key competencies should be developed to support training and delivery. As drivers for continuity of care in NSS pathways, patient navigators are particularly critical to safe communication in NSS pathways. Patient navigators can be recruited from a number of disciplinary backgrounds (e.g., nursing, radiography, administration) and relevant aspects of patient communication should be included in professional training.^50^

NSS pathways should provide both written materials and online resources for patients at the point of GP referral to improve organisational health literacy and support patient understanding. These materials should include information about the service, the clinical team and potential outcomes (see Table 3 for suggestions). Additional materials should be created to support patients' personal health literacy around understanding their results and incidental findings, including those with no follow‐up, and patients whose symptoms are not explained at discharge. These messages could be included in future National Institute for Health and Care Excellence guidance for suspected cancer in relation to NSS pathways.^51^

**Table 3:** Recommended messages for patient‐facing written and verbal communications about NSS pathways.

It is a cancer pathway The GP has made a referral due to NSS A clinical team that specialise in NSS will review your case You may have one or multiple investigations Several organ systems in your body are being investigated Results will come directly from the clinical team The scans may show other abnormalities in your body, these are often not serious, but they could also relate to diseases other than cancer for which appropriate treatment will be possible If they find a cancer, you will be redirected to a specialist team straight away If they find another disease which isn't cancer, they will also make sure that you are referred for appropriate care If they cannot find out what is causing your symptoms, they will give advice to the GP Your GP will not stop looking for the cause of your symptoms if they are persistent

Abbreviation: GP, general practitioner; NSS, non‐specific symptom.

## 4 CONCLUSIONS

The ability to understand and use health information to make informed decisions is a critical factor in patient engagement and safety, and cancer is one of the most complex and challenging diseases for patients to comprehend. This study has shown that patients with NSS experience a great deal of complexity in their care, even when being investigated by a specialist pathway. We have made recommendations for the key messages that should be communicated verbally and in written form to bring greater understanding to patients.

## AUTHOR CONTRIBUTIONS

**Georgia B. Black**: Conceptualisation; methodology; data curation; investigation; funding acquisition; writing—original draft; writing—review and editing; project administration; formal analysis. **Julie‐Ann Moreland**: Investigation; formal analysis; writing—review and editing. **Naomi J. Fulop**: Conceptualisation; methodology; supervision; writing—review and editing; funding acquisition; formal analysis. **Georgios Lyratzopoulos**: Conceptualization; funding acquisition; methodology; writing—review and editing; supervision. **Brian D. Nicholson**: Conceptualization; funding acquisition; methodology; writing—review and editing; supervision; formal analysis. **Katriina L. Whitaker**: Methodology; writing—original draft; writing—review and editing; formal analysis; supervision.

## CONFLICT OF INTEREST STATEMENT

The authors declare no conflict of interest.

## Supporting information

Supporting information.

Supporting information.

## Data Availability

The data that support the findings of this study are available on request from the corresponding author. The data are not publicly available due to privacy or ethical restrictions.
