# Supplementary material for: Personal and organisational health literacy in the non‐specific symptom pathway for cancer: An ethnographic study
Source: Health Expect. 2024 May 5;27(3):e14062. doi: 10.1111/hex.14062 (PMC11070181; doi:10.1111/hex.14062)
Supplement: Supplementary file 1 — Supporting information. [file HEX-27-e14062-s001.docx]

**Interview schedule:** **Professional hospital staff (RDC, other specialists)**

Drawing from O’Hara, R., Johnson, M., Hirst, E., Weyman, A., Shaw, D., Mortimer, P., Newman, C., Storey, M., Turner, J., Mason, S. and Quinn, T., 2014. A qualitative study of decision-making and safety in ambulance service transitions. *Health Services and Delivery Research*, *2*(56).

- Background information on the participants.
- Current roles/responsibilities, duration in employment.

What are the key parts of the pathway that you work in?

*Prompts:*

- *If the pathway works really well, what should happen?*
- *What would be the key signs that the pathway was or wasn’t working as it should?*

What are your main decisions in relation to patient care?

*Prompts:*

- *Triage*
- *Diagnostic tests etc*

What are the risks to patients associated with these decisions?

*Prompts:*

- *What influences your decisions about patient care?*
- *Where are the main risks and vulnerabilities in the pathway? (e.g. GP referral, transitions, patient DNA etc)*
- *What are your particular concerns about risks and patient safety?*
- *How are those risks mitigated?*

*(Consider resources e.g. skills/training; procedures/protocols; resources; pathways; equipment; task/service demands; ambulance service organisation and management).*

What are the patient’s responsibilities in the pathway?

*Prompts:*

- *What are the consequences if they are not met?*
- *What type of information do patients need to know?*
- *How is it communicated?*

How do other individuals and services *inside* the hospital affect the safety of the pathway?

How do services and organisational bodies *outside* the hospital affect the safety of the pathway?

What could be done differently?

Anything else relevant to the risk(s) associated with decisions about patient care?
