# Supplementary material for: Personal and organisational health literacy in the non‐specific symptom pathway for cancer: An ethnographic study
Source: Health Expect. 2024 May 5;27(3):e14062. doi: 10.1111/hex.14062 (PMC11070181; doi:10.1111/hex.14062)
Supplement: Supplementary file 2 — Supporting information. [file HEX-27-e14062-s002.docx]

**Interview schedule: Patients and carers**

Drawn from Wengraf, T. and Chamberlayne, P., 2006. Interviewing for life-histories, lived situations and personal experience: The Biographic-Narrative Interpretive Method (BNIM). *Short Guide to BNIM interviewing and interpretation*.

- Background information

Biographical interview section: Please tell me the story of how you (/the person you care for) came to be referred to this service, all the experiences and events which were important for you, up to now.

- - *Start wherever you like*
  - *Please take the time you need*
  - *I’ll listen first, I won’t interrupt*
  - *I’ll just take some notes in case I have any further questions for after you’ve finished telling me about it all*

Elaboration section: Researcher to make note of particular ‘cue-phrases’ in thematic order suggested by participant and return to each in turn for more information.

*Prompts:*

- *Open elaboration e.g. “can you tell me more about X?”*
- *Focussed elaboration e.g. “can you think of a moment when you felt X particularly?”*
- *Appraisal “how did you make sense of X?” / “what were you thinking at the time?” / “what do you think now?”*

Future casting section: what will happen now?

Prompts:

- *What did the HCP tell you?*
- *What do you need to do?*
- *What will happen at that meeting?*
- *What do you know about your symptoms now?*

Additional questions about anything the researcher witnessed or heard about during shadowing.

Is there something else that you wanted to talk about?
